# Personalized Acute Upper GI Bleeding Diagnostics for Patients at Highest Risk for Endoscopy: Real-World Experience of a Novel, Binary, Bedside Gastric Blood Detection Device

**DOI:** 10.3390/jpm15120573

**Published:** 2025-11-28

**Authors:** Hadi Khaled Abou Zeid, Manik Aggarwal, Jad P. AbiMansour, Miranda Hamlin, Yara Salameh, Karl Akiki, Andrew C. Storm

**Affiliations:** 1 Division of Gastroenterology and Hepatology, Mayo Clinic School of Medicine, 200 1st St SW, Rochester, MN 55902, USAhamlin.miranda@mayo.edu (M.H.);; 2 Department of Internal Medicine, Cooper University Health Care, Rochester, NJ 08103, USA

**Keywords:** endoscopy, upper gastrointestinal bleed, personalized medicine, triage, innovation

## Abstract

**Background:** Upper gastrointestinal bleeding (UGIB) is a common medical emergency associated with significant morbidity, mortality, and healthcare costs. Many patients undergo early endoscopy despite the absence of active bleeding. PillSense is a novel Food and Drug Administration (FDA)-cleared ingestible capsule that rapidly detects the presence of blood in the upper GI tract and may optimize triage decisions. **Methods:** We conducted a retrospective study evaluating the impact of PillSense on the management of suspected UGIB in an academic center. The primary outcome was the association between capsule results and clinical decision-making, including endoscopy deferral, prioritization, outpatient scheduling, and airway protection. Secondary outcomes included transfusion requirements, time-to-endoscopy, endoscopic intervention, and 30-day adverse events. **Results:** A total of 28 patients (mean age 64.4 ± 17.9 years, 82.7% male) were included. Compared with negative results, positive results were associated with higher transfusion requirements (median 3 (IQR 3–6) vs. 2 (IQR 1–3.25) units; *p* = 0.041) and shorter time-to-endoscopy (median 0.2 (IQR 0.01–1) vs. 2 (IQR 1–15.5) days; *p* = 0.017). In high-risk for sedated endoscopy patients, negative results were associated with EGD deferral in 53.8%, with no subsequent adverse events within 30 days. Endoscopic intervention was performed in 62.5% of positive-result patients versus 9.5% of negative-result patients. **Conclusions:** The PillSense results were associated with differences in triage and management of high-risk patients with suspected UGIB. Its rapid, accurate, and non-invasive results may reduce unnecessary urgent endoscopy procedures, improve resource utilization, and enhance patient safety, particularly in the highest-risk populations.

## 1. Introduction

Upper gastrointestinal bleeding (UGIB) occurs in 48 to 160 cases per 100,000 individuals and accounts for roughly 300,000 hospital admissions per year in the United States alone, with over USD 3.3 billion in direct hospital costs [1]. Its mortality rate has been estimated to be around 5–10% [2]. Patients with significant bleeding often present with hemodynamic instability, requiring rapid resuscitation, transfusion, and hospitalization [1].

Current management strategies for suspected UGIB combine assessment of the patient’s hemodynamic status with the use of risk stratification tools to establish diagnosis and provide therapy [1]. The Glasgow-Blatchford Score (GBS) is a pre-endoscopic score designed to predict the need for clinical intervention (such as blood transfusion, endoscopic therapy, or surgery) in UGIB. A GBS ≤1 indicates very low risk <1% of requiring intervention or dying and current guidelines recommend that such patients may be safely discharged with outpatient follow-up if no other concerning features are present. In contrast, patients with higher scores necessitate in-hospital care and endoscopy. The Rockall score incorporates pre-endoscopic clinical parameters (age, shock, comorbidities) and endoscopic findings (diagnosis and signs of active hemorrhage) to estimate risk of rebleeding and mortality. Other tools like AIMS65 (which accounts for Albumin, INR, Mental status, Systolic BP, and age ≥ 65) and ABC (Age, Blood tests, and Co-morbidities) have also been developed primarily to predict in-hospital mortality. Comparative studies indicate that AIMS65 outperformed both GBS and pre-endoscopic Rockall Score (pRS) in predicting in-hospital mortality, though all scores demonstrated only modest accuracy for identifying the need for clinical interventions in high-risk patients. Moreover, a more recent analysis reported that all tools (GBS, AIMS65, pRS, and ABC) had limited discriminative ability for predicting in-hospital clinical interventions, with AUROC values below 0.65. As a result, current guidelines from the international consensus, American College of Gastroenterology (ACG), and European Society of Gastrointestinal Endoscopy (ESGE) only endorse GBS with a threshold of ≤1 to identify patients who can be safely managed as outpatients [3].

Ultimately, the majority of patients receive diagnostic Esophagogastroduodenoscopy (EGD) within 24 h [4]. A large UK cohort study involving 47,481 EGDs performed for suspected UGIB showed that therapeutic endoscopic interventions were required in only 14.8–20% of cases [5]. Moreover, this practice often overburdens endoscopy services [6,7]. Even though EGD is generally considered low risk for adverse events with a reported complication rate 0.1–0.3%, specific populations, such as critically ill patients and patients with severe cardiopulmonary comorbidities, have higher risks of cardiopulmonary and other procedural and anesthetic-related adverse events [8,9,10]. These findings highlight an important gap in current UGIB management. While early endoscopy improves patient outcomes, it may not always achieve an optimal balance between risks and benefits across all patient subgroups [3]. Some patients with high-risk for sedated endoscopy may undergo EGDs that yield no endoscopic interventions. This underscores the need for more personalized UGIB triage strategies.

PillSense, a novel FDA-cleared (De Novo, DEN220065) ingestible capsule (Enterasense; Galway, Ireland), offers an innovative approach to triage in suspected UGIB [11]. In a prospective clinical trial, the capsule demonstrated 92.9% sensitivity, 90.6% specificity, and 97.8% negative predictive value for detecting blood in the upper GI tract, with no adverse events and natural passage in all patients [7]. The system provides a binary result (‘Blood Detected’ vs. ‘No Blood Detected’) without the need for sedation or endoscopic expertise [11]. By rapidly identifying patients without active bleeding, PillSense has the potential to reduce unnecessary urgent endoscopies, streamline resource use, and personalize acute UGIB management, ensuring that high-risk patients receive timely intervention while low-risk patients avoid invasive procedures [7].

In this study, we outline a real-world experience in personalizing the management of acute UGIB using PillSense in an academic center for patients in the highest-risk category for undergoing sedated endoscopy, including patients admitted to the intensive care unit or who were determined to have high risk for sedation-related complications. We also highlight the different use cases of PillSense across different clinical practice settings. Our aim is to further enhance the use of this available technology for the personalized diagnosis and care of patients with suspected acute UGIB.

## 2. Materials and Methods

### 2.1. Study Design and Patients

We conducted a retrospective study to assess the clinical application of the blood detection capsule in patients with clinical signs of acute GI bleed between January 2024 and July 2025 at our single tertiary care center. Patients were considered high-risk for anesthesia-related complications if they had severe cardiopulmonary disease, were classified as ASA IV–V, demonstrated hemodynamic instability, or were admitted to the intensive care unit (ICU). All ICU patients had been admitted for other causes and subsequently developed signs of UGIB during hospitalization. Patients with GI tract obstructions, motility disorders, dysphagia, pregnancy, or implanted electromedical devices were excluded, as per the device’s indications for use. The capsule was used by gastroenterologists to guide the management of suspected acute UGIB.

### 2.2. Device Description

**Figure 1:**
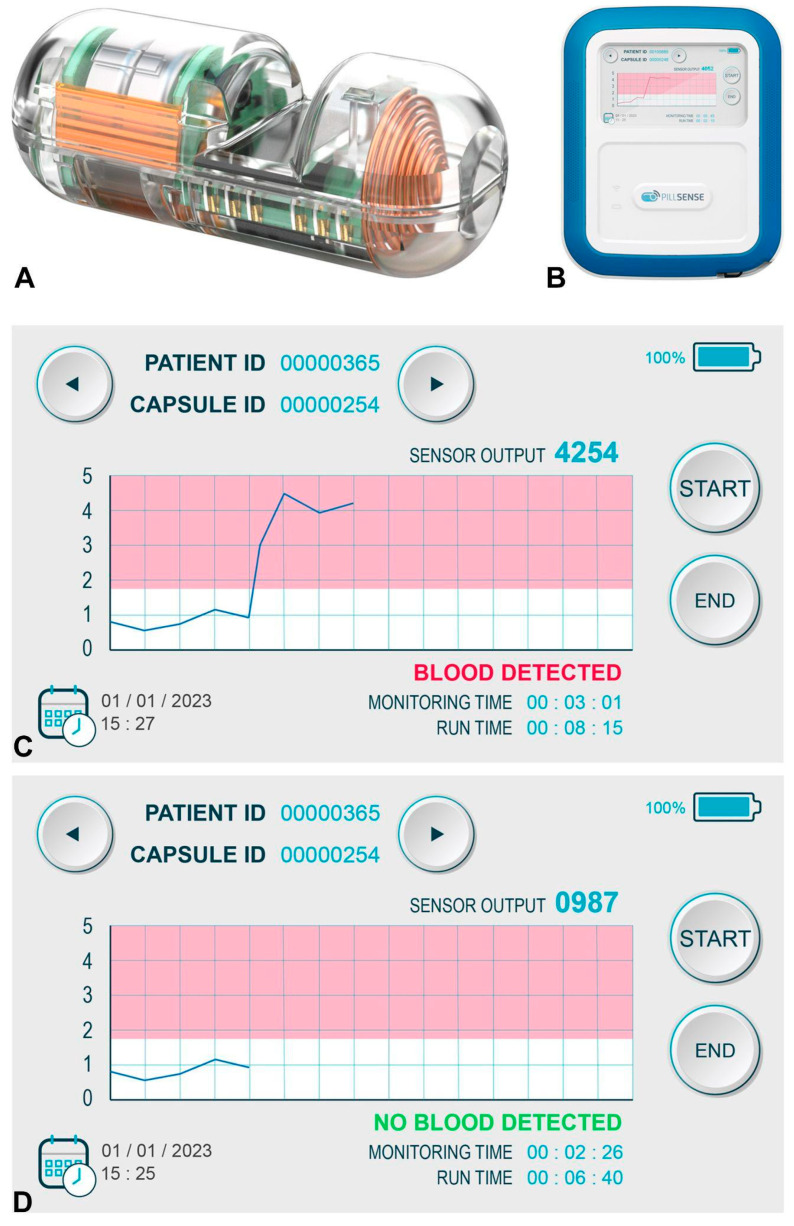
Components and output of the PillSense system. (**A**) The ingestible blood detection capsule. (**B**) The external receiver device used to collect and process signals. (**C**) Example of a positive capsule result. (**D**) Example of a negative capsule result.

The PillSense system included an ingestible capsule and a receiver. The capsule detected the presence of blood by analyzing light absorption at various wavelengths. The receiver wirelessly received data from the capsule and displays a simple binary result: “Blood Detected” or “No Blood Detected”. The entire process took approximately 10 min and did not require sedation or endoscopic procedure or image interpretation expertise. The device was operated by residents or clinical fellows. No patients underwent repeat capsule testing (Figure 1).

### 2.3. Outcomes

Patients were stratified into two groups according to capsule findings (positive vs. negative). The primary outcome was the impact of PillSense on clinical decision-making in suspected UGIB, including its influence on EGD deferral, prioritization, outpatient scheduling, and airway protection. Secondary outcomes included transfusion requirements, time-to-endoscopy, rate of endoscopic intervention, and 30-day adverse events. 30-day outcomes including rebleeding, readmission, and mortality were determined through review of the institutional EHR, which captures inpatient and outpatient encounters within our health system as well as encounters from other institutions that also use the EPIC platform. Rebleeding was defined as recurrent overt GI bleeding (melena, hematemesis, hematochezia, or coffee-ground emesis) documented after receiving PillSense. Readmission was defined as any unplanned hospitalization or Emergency Department visit within 30 days. Mortality was defined as all-cause death within 30 days. No patients were discharged to outside facilities, but events occurring outside EPIC institutions could theoretically be missed.

### 2.4. Statistical Analysis

Continuous variables were presented as mean (SD) or median (IQR), and categorical variables were expressed as percentages. Comparisons between groups were performed using the Wilcoxon rank-sum test, while categorical variables were compared using χ^2^ or Fisher’s exact test as appropriate, with a *p*-value < 0.05 considered statistically significant. Statistical analyses were performed by using R version 4.3.2 (R Foundation for Statistical Computing, Vienna, Austria).

Given the exploratory nature and small sample size of this study, confidence-interval estimates should be interpreted cautiously, as the analyses may be underpowered to detect smaller effect sizes.

### 2.5. Ethics

This study was reviewed and waived by the Institutional Review Board of Mayo Clinic (Rochester, MN). Given the retrospective nature of the study, the requirement for informed consent was waived. All procedures were conducted in accordance with the ethical principles of the Declaration of Helsinki and relevant institutional guidelines for clinical research. This study received no funding. The device manufacturer had no role in study design, data collection, data analysis, interpretation, or manuscript preparation.

## 3. Results

**Figure 2:**
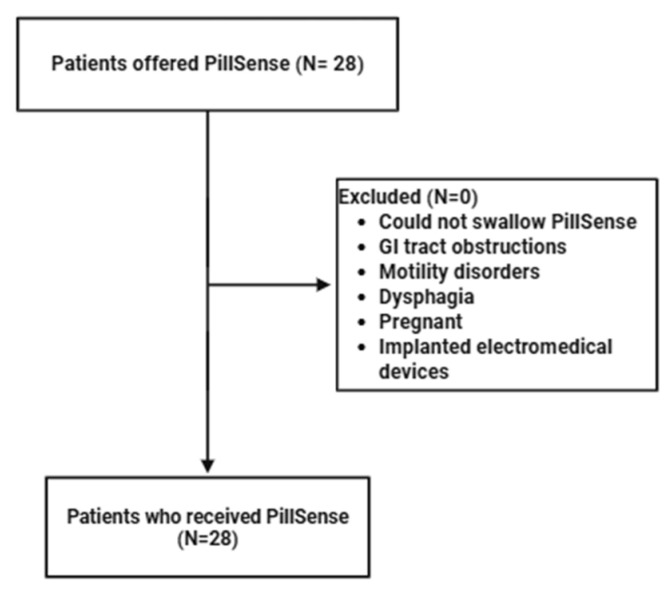
STROBE-style flow diagram.

A total of 28 patients with a mean (SD) age of 64.4 (17.9), 82.7% males were included (Figure 2). No patients were excluded. The median time from presentation to capsule ingestion was 1.5 h (IQR 0.5–5.0). Eight patients had a positive capsule result. Patients with a positive capsule result were associated with significantly higher transfusion requirements (median 3 units (IQR 3–6) vs. 1 unit (IQR 0–2); 95% CI −11.2–38.9 vs. 0.6–3.2; *p* = 0.025) and shorter time-to-endoscopy (median 0.2 days (IQR 0.01–1) vs. 2 days (IQR 1–21); 95% CI −0.7–3 vs. −1–21); *p* = 0.02). There were not statistically significant differences in GBS (13.2 ± 5.85 vs. 11.4 ± 4.03; *p* = 0.13) and hospital stay (median 5.5 days (IQR 3–12) vs. 7 days (IQR 4–21); *p* = 0.43) between the 2 groups. Among patients with positive capsule results 5/8 (62.5%) had active UGIB and required endoscopic management. Among patients with negative capsule results, 2/21 (9.5%) had signs of active UGIB required endoscopic intervention.

Regarding device adverse events, there was 1 case of capsule retention in a patient with a prior history of radical cystoprostatectomy, pelvic and retroperitoneal lymph node dissection, and ileal conduit. The capsule was retrieved endoscopically via colonoscopy without complications. No other adverse events such as nausea, vomiting, or device intolerance were observed.

### 3.1. Patients at High-Risk for Sedated Endoscopy

A total of 20 patients with mean (SD) age 64.6 (16.2) years and 85% male were included (Table 1). Of these, 13 (65%) were in a critical care setting and 7 (35%) were regarded as high-risk for sedation-related complications; 15/20 (75%) had melena, 5/20 (20%) had hematemesis, and 2/20 (10%) had syncope; and 12 patients were hemodynamically unstable. PillSense results were positive in 7 patients. In this group, EGD was prioritized in 5/7 (71.4%), and 1 patient was intubated prior to endoscopy for airway protection because we expected large volume of blood due to the immediate detection of blood by the PillSense capsule. Among patients with negative capsule results, EGD was deferred in 7/13 (53.8%) with mean (SD) GBS of 10.3 (4.2). Among patients in which upper endoscopy was deferred, none required subsequent endoscopy, experienced readmission for gastrointestinal bleeding, or mortality within 30 days.

### 3.2. Inpatients at Standard Risk for Sedated Endoscopy

Eight hospitalized patients with mean age (SD) 62.1 (21.7) years, 87.5% males, and standard risk for sedation-related complications were included (Table 2). PillSense was positive in 1 patient, in whom EGD was prioritized and performed on the same day. Among patients with negative PillSense, EGD was deferred in 4/7 patients with mean GBS (SD) 10.75 (1.89). Moreover, colonoscopy without EGD was recommended in one patient with hematochezia after ruling-out UGIB with negative capsule results, and outpatient EGD/Colonoscopy were recommended for anemia work-up in another patient. Among patients in which upper endoscopy was deferred, none required subsequent endoscopy, experienced readmission for gastrointestinal bleeding, or mortality within 30 days.

## 4. Discussion

In this real-world retrospective analysis, we found that PillSense results (positive versus negative) were significantly associated with UGIB clinical severity and differences in resource utilization and clinical decision-making. Patients with a positive capsule result were associated with significantly higher transfusion requirements and a shorter time-to-endoscopy, compared to those with negative results. In addition, the capsule results appeared to influence triage and clinical decision-making for patients with upper GI bleeding such as decisions for EGD deferral, EGD prioritization, outpatient EGD, and even airway management. Together, these findings indicate that PillSense may support personalized management decisions in suspected UGIB.

Our study is the first to describe the real-world impact of PillSense on clinical decision-making for suspected UGIB across multiple care settings, including the ICU and inpatient wards. Our findings build upon the previously published clinical trials that demonstrated the safety and diagnostic accuracy of PillSense [7].

When interpreted alongside clinical findings, the capsule result may support physicians’ triage for UGIB and guide management plans. In fact, EGD was deferred in over 50% of patients determined to be high-risk for anesthesia-related complications with negative capsule results. This is particularly important in such high-risk populations, where the procedural and anesthetic risks of urgent EGD may outweigh potential benefits if active or recent bleeding is absent. In the inpatient wards, negative PillSense was associated with clinical decisions to delay or defer EGD and even redirect the diagnostic approach toward the lower GI tract or anemia workup, suggesting that it may safely narrow down the diagnostic differential and reduce unnecessary and costly inpatient upper endoscopy procedures.

On the other hand, positive capsule results were associated with appropriate escalation of care including prioritization of EGD with shorter time-to-endoscopy and pre-endoscopy intubation in anticipation of high-volume bleeding. This phenomenon of decreasing time to endoscopy was also seen with the now discouraged practice of nasogastric tube lavage, which was discontinued due to poor sensitivity and specificity of that test [12]. It is also important to mention that positive capsule results were associated with more endoscopic interventions compared to negative capsule results (62.5% vs. 9.5%) despite no statistically significant difference in GBS between the 2 groups.

PillSense offers several advantages over traditional risk stratification score and other diagnostic procedures. A retrospective study evaluated the clinical utility of GBS in 211 patients with suspected UGIB. Although GBS showed promising findings in the stratification of patients based on severity with 0% 30-day mortality in the low-risk population and 3% in the high-risk population, it tended to overestimate the need for endoscopy with only 31.8% high-risk patients requiring endoscopic interventions [13]. In our cohort, high-risk for sedated endoscopy patients with positive capsule results had a slightly higher mean GBS compared with the negative group (15.1 vs. 11.9, *p* = 0.047), however, both cohorts were within the high-risk for acute GI bleeding range. This suggests that the observed GBS difference may not reflect a clinically meaningful difference in bleeding probability, underscoring the value of PillSense in providing direct, real-time evidence of active bleeding to complement risk stratification tools. Similarly, video capsule endoscopy, while useful in certain contexts, requires specialized image interpretation and does not provide rapid binary results [7]. Even though EGD is the gold standard for UGIB diagnosis and treatment, only 14.8–20% of suspected UGIB require endoscopic treatment [5]. This means the majority of urgent endoscopies consume significant hospital resources, procedure rooms, anesthesia teams, ICU beds, without direct therapeutic benefit. PillSense can provide rapid non-invasive and accurate results that could potentially stratify patients based on the real-time evidence of acute GI bleeding, allowing physicians to either prioritize or avoid invasive interventions. This will also be specifically beneficial for community and rural hospitals, where it could guide transfer decisions and reduce unnecessary escalation of care.

**Figure 3:**
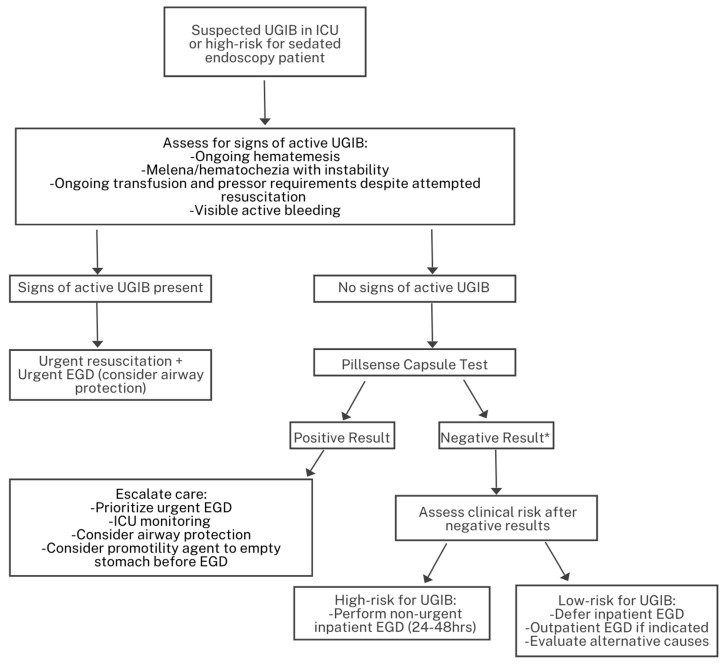
Proposed algorithm for the triage of suspected UGIB in ICU patients or those at high risk for sedated endoscopy. * Patients with negative PillSense results should undergo continued clinical monitoring. Persistent hemodynamic instability, ongoing hematemesis or melena, or a decline in hemoglobin despite a negative result should prompt early endoscopy or repeat diagnostic evaluation.

Based on these findings, we propose a practical triage algorithm (Figure 3) that integrates PillSense results with clinical assessment for patients at high risk for sedation-related complications. This algorithm provides a structured approach for escalation or de-escalation of care: positive results support urgent endoscopy, airway protection, and consideration of promotility agents, while negative results allow for safe deferral or outpatient management depending on residual clinical risk. In patients with negative PillSense results but ongoing clinical concern, management should emphasize close monitoring and reassessment rather than immediate reassurance. Persistently unstable hemodynamics, declining hemoglobin, or continued hematemesis or melena should prompt early endoscopy or repeat diagnostic evaluation. By combining real-time evidence of bleeding with bedside assessment, this framework aims to reduce unnecessary urgent endoscopies while ensuring timely intervention for those most likely to benefit. While this algorithm reflects our real-world experience, it needs further prospective validation in larger multicenter cohorts before implementation.

In our cohort, two patients with negative PillSense results required endoscopic intervention, including one patient with active UGIB from duodenal ulcers. Potential explanations include intermittent bleeding that ceased before capsule ingestion or rapid capsule transit past the bleeding site. Because PillSense is not validated for small-bowel or lower GI tract bleeding, persistent anemia or ongoing overt bleeding despite a negative PillSense should prompt further evaluation of the small bowel Moreover, PillSense does not differentiate between variceal and non-variceal sources, and a negative result does not preclude subsequent rebleeding. These limitations highlight the need to interpret results within the broader clinical context, including evolving symptoms, hemodynamic status, and laboratory trends. In line with current guidelines recommending endoscopy within 24 h for most UGIB presentations, PillSense should be regarded as a complementary triage tool to help prioritize care, but not as a replacement for timely EGD. Device-related adverse events were rare. Capsule retention occurred in only one patient highlighting the importance of careful patient selection and the need to account for altered anatomy that may affect capsule transit.

The strengths of our study include the incorporation of real-world management outcomes from a tertiary care center, analysis across different care environments, and the inclusion of a clinically diverse patient population. Limitations include the retrospective design with potential selection bias, the small sample size, and the single-center setting, which limit generalizability and preclude exclusion of residual confounding by other factors (GBS, hemodynamic instability, ASA class, and antithrombotic use). As the use of PillSense was limited to patients in whom it was applied during routine care, we cannot report the total number of suspected UGIB cases during the study period, which reduces representativeness and generalizability. Although outcome ascertainment was performed within EPIC, which captures encounters across multiple health systems, events occurring outside EPIC institutions could theoretically be missed.

Future research should focus on multicenter prospective validation of these findings and direct comparisons between capsule-guided care and traditional triage strategies. Cost-effectiveness analyses will be essential to define the value proposition of incorporating PillSense into routine clinical workflows.

## 5. Conclusions

In this real-world analysis, the blood-detecting capsule demonstrated a meaningful impact on UGIB triage and management. The PillSense results were associated with differences in clinical decision-making, transfusion requirements, and time-to-endoscopy, supporting both escalation and de-escalation of care across varied clinical settings. By rapidly providing accurate non-invasive results, it may offer clear advantages over traditional risk scores and other diagnostic tools, potentially reducing unnecessary urgent endoscopies, optimizing resource allocation, and enhancing individualized care and patient safety, particularly in high-risk populations.

This study was limited by its retrospective single-center design, small sample size, and potential selection bias, which may limit generalizability. Larger multicenter prospective studies are warranted to validate these findings, evaluate the impact of PillSense-guided triage on clinical outcomes, and determine the cost-effectiveness of integrating this technology into standard UGIB management pathways.

## Figures and Tables

**Table 1:** High-risk patients for Sedated Endoscopy.

	Positive Capsule	Negative Capsule	*p*-Value
Age, Mean (SD)	57.3 (22.1)	68.1 (10.6)	0.259
Male, *n* (%)	7 (100%)	9 (69.2%)	0.245
BMI, Mean (SD)	23.5 (5.5)	30 (4.9)	0.024
ASA IV-V, *n* (%)	2 (28.6%)	7 (53.8%)	0.387
Liver Disease, *n* (%)	4 (57.1%)	2 (15.4%)	0.140
CAD, *n* (%)	1 (14.3%)	5 (38.5%)	0.347
Heart Failure, *n* (%)	2 (28.6%)	7 (53.8%)	0.387
Valvular Disease, *n* (%)	2 (28.6%)	2 (15.4%)	0.585
Critical Care Setting, *n* (%)	6 (85.7%)	7 (53.8%)	0.315
GBS, Mean (SD)	15.1 (2.5)	11.9 (4.2)	0.047
Melena, *n* (%)	6 (85.7%)	8 (61.5%)	0.347
Hematemesis, *n* (%)	2 (28.6%)	1 (7.7%)	0.271
Coffee-ground emesis, *n* (%)	0 (0%)	2 (15.4%)	0.515
Anemia, *n* (%)	5 (71.4%)	8 (61.5%)	1.000
Hematochezia, *n* (%)	1 (14.3%)	6 (46.2%)	0.198
Syncope, *n* (%)	1 (14.3%)	1 (7.7%)	1.000
Hemodynamic Instability, *n* (%)	6 (85.7%)	6 (46.2%)	0.170
Hemoglobin, Mean (SD)	7.5 (1.3)	8.5 (2.4)	0.242
INR, Mean (SD)	1.4 (0.3)	1.4 (0.8)	1.000
Antiplatelets, *n* (%)	3 (42.9%)	8 (61.5%)	0.659
Anticoagulation, *n* (%)	2 (28.6%)	7 (53.8%)	0.387

BMI: body mass index; ASA: American Society of Anesthesiologists physical status classification; CAD: coronary artery disease; GBS: Glasgow-Blatchford score; INR: international normalized ratio.

**Table 2:** Inpatient Standard-risk Patients for Sedated Endoscopy.

	Positive Capsule	Negative Capsule
Age, Mean (SD)	18 *	67.6 (15.1)
Male, *n* (%)	1 (100%)	6 (85.7%)
BMI, Mean (SD)	31.7 *	27.1 (4.7)
ASA IV-V, *n* (%)	0 (0%)	3 (42.9%)
Liver Disease, *n* (%)	0 (0%)	2 (28.6%)
CAD, *n* (%)	0 (0%)	2 (28.6%)
Heart Failure, *n* (%)	0 (0%)	1 (14.3%)
Valvular Disease, *n* (%)	0 (0%)	0 (0%)
GBS, Mean (SD)	0 *	10 (3.1)
Melena, *n* (%)	0 (0%)	6 (85.7%)
Hematemesis, *n* (%)	1 (100%)	0 (0%)
Coffee-ground emesis, *n* (%)	1 (100%)	0 (0%)
Anemia, *n* (%)	0 (0%)	3 (42.9%)
Hematochezia, *n* (%)	0 (0%)	1 (14.3%)
Syncope, *n* (%)	0 (0%)	1 (14.3%)
Hemodynamic Instability, *n* (%)	0 (0%)	1 (14.3%)
Hemoglobin, Mean (SD)	15.4 *	8.5 (2.5)
INR, Mean (SD)	NA	1.3 (0.5)
Antiplatelets, *n* (%)	0 (0%)	5 (71.4%)
Anticoagulation, *n* (%)	0 (0%)	3 (42.9%)

BMI: body mass index; ASA: American Society of Anesthesiologists physical status classification; CAD: coronary artery disease; GBS: Glasgow-Blatchford score; INR: international normalized ratio, NA: Not applicable. * For groups with only one patient, individual values are shown.

## Data Availability

The original contributions presented in this study are included in the article. Further inquiries can be directed to the corresponding authors. H.K.A.Z., M.A., Y.S., and A.C.S. had full access to all raw data used in this study. All statistical analyses were performed by H.K.A.Z. and independently verified internally by Y.S., and A.C.S. The data are not publicly available due to privacy restrictions.

## Abbreviations

The following abbreviations are used in this manuscript:

UGIB	Upper Gastrointestinal Bleeding
EGD	Esophagogastroduodenoscopy
ICU	Intensive Care Unit
GBS	Glasgow-Blatchford Score
ASA	American Society of Anesthesiologists
BMI	Body Mass Index
INR	International Normalized Ratio
